# Undiagnosed Impaired Glucose Tolerance and Type-2 Diabetes in Acute Myocardial Infarction Patients: Fequency, Characteristics and Long-Term Mortality

**DOI:** 10.3389/fcvm.2022.869395

**Published:** 2022-04-25

**Authors:** Timo Schmitz, Eva Harmel, Margit Heier, Annette Peters, Jakob Linseisen, Christa Meisinger

**Affiliations:** ^1^Chair of Epidemiology, University Hospital Augsburg, University of Augsburg, Augsburg, Germany; ^2^Department of Cardiology, Respiratory Medicine and Intensive Care, University Hospital Augsburg, Augsburg, Germany; ^3^KORA Study Centre, University Hospital of Augsburg, Augsburg, Germany; ^4^Helmholtz Zentrum München, Institute for Epidemiology, Neuherberg, Germany; ^5^Chair of Epidemiology, Medical Faculty, Institute for Medical Information Processing, Biometry and Epidemiology, Ludwig-Maximilians-Universität München, Munich, Germany; ^6^German Center for Diabetes Research (DZD), Neuherberg, Germany

**Keywords:** prediabetes, HbA1c, myocardial infarction, long-term mortality, undiagnosed diabetes

## Abstract

**Background:**

In this study we investigated the prevalence of undiagnosed impaired glucose tolerance and type-2-diabetes (T2D) among patients with acute myocardial infarction (AMI) and prospectively analyzed whether these patients have a higher long-term mortality.

**Methods:**

The analysis was based on 2,317 AMI patients aged 25–84 years from the population-based Myocardial Infarction Registry Augsburg, recruited between 2009 and 2014 and followed-up until 2019 (median follow-up time 6.5 years [IQR: 4.9–8.1]). AMI patients with a diagnosis of diabetes were divided into a high (>7.0%) and a low HbA1c group (≤7.0%) according to HbA1c values at admission. The remaining patients (without known diabetes) were grouped into normal (<5.7%), elevated (5.7–6.4%), and high (≥6.5%) HbA1c groups. In a multivariable-adjusted COX regression analysis, the association between HbA1c groups and long-term mortality was investigated. Linear regression models were used to identify AMI patients with elevated HbA1c values by means of personal characteristics.

**Results:**

At admission, 29.5% of all patients reported a diagnosis of diabetes. Of all patients without known diabetes, 5.4% had HbA1c values of ≥ 6.5 and 37.9% had HbA1c values between 5.7 and 6.4%. The fully adjusted Cox regression model showed a non-significant trend toward higher long-term mortality for AMI patients with increased HbA1c values (HbA1c 5.7–6.4% HR: 1.05 [0.79–1.38], HbA1c > 6.5% HR: 1.34 [0.77–2.31]). A linear regression model including the variables admission serum glucose, BMI, age, sex and type of infarction (STEMI, NSTEMI) showed only poor prediction of HbA1c values (*R*^2^: 11.08%).

**Conclusion:**

A fairly high number of AMI patients without known diabetes have elevated HbA1c values. Though we could not prove a higher risk of premature mortality in these patients, early detection and adequate therapy might lead to reduced diabetes-associated complications and improve long-term outcomes.

## Introduction

The prevalence of diabetes mellitus, and in particular type 2 diabetes, is increasing not only in Europe, but also in most other parts of the world at an alarming rate (1). It is known to be a major risk factor for several diseases, including coronary artery diseases and acute myocardial infarctions (AMI) (2). Diabetes mellitus not only increases the risk of experiencing such an event, but also affects the overall prognosis and outcome. Therefore, it is essential to detect and treat diabetes as early as possible. However, the number of undetected diabetes cases worldwide is as high as the number of diagnosed cases, which presents a major challenge (1). What’s more, prior studies have also suggested a very high prevalence of undiagnosed diabetes in patients with AMI (3–10). Nevertheless, most of these studies were not population-based and thus results might be biased; furthermore, the consequences that “hidden” diabetes in AMI patients may have on long-term mortality has rarely been investigated. Therefore, the aim of this study is to give a reliable estimation of the prevalence of undiagnosed diabetes among AMI patients using population-based data with consecutive enrollment from the Augsburg Myocardial Infarction Registry. In addition, this study examines the association between diabetes/HbA1c values and long-term mortality in AMI patients. Finally, we tried to identify risk factors for elevated HbA1c values in AMI patients.

## Materials and Methods

### Study Population

We used data from the population-based Augsburg Myocardial Infarction Registry (previously the KORA Myocardial Infarction Registry), which was established in 1984 as a part of the MONICA-project (Monitoring Trends and Determinants in Cardiovascular disease) (11). The study area consists of the city of Augsburg, Germany, and the two adjacent counties comprising a total of approximately 680,000 inhabitants. Patients aged between 25 and 84 years who were admitted to one of the eight hospitals in the study area due to AMI were registered. More detailed information on case identification, diagnostic classification of events and quality control of the data can be found in previous publications (11, 12). For the present study, only patients with AMI who were admitted to the University Hospital Augsburg between 2009 and 2014 were included, since blood samples from AMI patients were collected solely in this hospital. All study participants gave written, informed consent. Methods of data collection have been approved by the ethics committee of the Bavarian Medical Association (Bayerische Landesärztekammer), and the study was performed in accordance with the Declaration of Helsinki.

Participants with missing information on diabetes, HbA1c and relevant covariables (*n* = 501) were excluded. The final study population consisted of 2,317 patients with AMI. For the total of 1,894 patients who survived the first 28 days after the infarction, additional information on long-term survival was available. These patients were followed-up for a median time of 6.5 (IQR: 4.9–8.1) years.

### Data Collection

Trained study nurses interviewed the participants during the hospital stay using a standardized questionnaire. In order to confirm the information provided by the patients and to collect additional information, the patients’ medical chart was reviewed. Demographic data and data on cardiovascular risk factors, medical history, comorbidities (including diabetes) and medication before and during hospital stay as well as at discharge was collected for each patient. Furthermore, admission ECG presentation, in-hospital course of the disease and several laboratory parameters including glucose measurement were determined. During the interview, all patients were asked if they suffer from diabetes mellitus. The presence of diabetes mellitus in a patient was additionally extracted from the medical chart. The patient was assigned to the non-diabetes group when there was neither an indication for existing diabetes mellitus (including a previous instance of disturbed glucose tolerance) in the interview nor in the medical chart. All other cases were assigned to the diabetes group. HbA1C values were not used for diabetes grouping. For this study, we did not make a distinction between diabetes mellitus type 1 and type 2.

One variable was generated to register whether it was known that a patient received all four evidence-based medications (EBM) at discharge or not (antiplatelet drug, ACE blockers/ATII antagonist, beta-blockers, statins). Any known cardiological in-hospital complication including cardiogenic shock, left ventricular decompensation, bradycardia, in-hospital reinfarction, ventricular tachycardia and ventricular fibrillation was summarized in one variable (yes/no).

Between 2009 and 2014, plasma samples were collected from AMI cases during the hospital stay and frozen at −80°C.

#### Clinical Chemistry Measurement

HbA1c values were measured in stored samples with a reverse-phase cation-exchange high-pressure liquid chromatography (HPLC) method (Analyzer HA 8160; Menarini, Florence, Italy). All other blood parameters were measured in-house during the hospital stay of the patients as part of the regular diagnosis and routine treatment.

#### Outcome

The endpoint used in this study was long-term all-cause mortality. Mortality was ascertained by regularly checking the vital status of all registered persons of the MI registry with data from the population registries. Death certificates were obtained from local health departments.

#### Grouping According to Known Diabetes and HbA1c Values

According to HbA1c values at admission, patients with known diabetes were divided into two groups by either high or low HbA1c values (>7.0 vs. ≤7.0% respectively). The remaining patients without known diabetes were grouped into a normal HbA1c group (<5.7%), an elevated group (5.7–6.4%) and a high group (≥6.5%).

### Statistical Analysis

For the comparison of categorical variables, Chi-square tests were performed and the results were presented as absolute frequencies with percentages. For normally-distributed continuous variables, Student’s t-test was used. For continuous variables that were not normally distributed, we used non-parametric tests. The results were presented as medians with inter-quartiles ranges.

#### Cox Regression Models

To further examine the association between diabetes/HbA1c values and long-term mortality, several Cox regression models were calculated. In order to focus on long-term mortality exclusively, we eliminated all patients who died within the first 28 days after infarction. First, a model including only the diabetes/HbA1c group was calculated. A second model included sex and age. According to a literature review, the final model was adjusted for the following confounders: sex, age, typical chest pain symptoms, diabetes, smoking, hyperlipidemia, hypertension, left-ventricular EF ≤ 30%, impaired renal function (according to eGFR), BMI, systolic blood pressure, PCI, bypass surgery, lysis therapy, EBM, and statin prescription at discharge. The proportional hazards assumption was checked by plotting the Schoenfeld residuals against time and searching for any visible correlation. Additionally, a test was performed to check for a significant correlation of the Schoenfeld residuals with time and consequently a violation of the proportional hazard assumption.

#### Linear Regression Models

In order to identify predictors of elevated HbA1c values among patients without known diabetes, linear regression models were calculated. For each model, the dependent outcome variable was HbA1c. One linear model was calculated for each of the following variables: BMI, age, glucose at admission, sex and type of AMI. Finally, a model including all five variables was calculated to predict HbA1c values. *R*^2^ values were used to measure the goodness of adaptation.

All statistical analyses were carried out with the R program version 4.1.0. A *p*-value of < 0.05 was considered as statistically significant.

## Results

**FIGURE 1:**
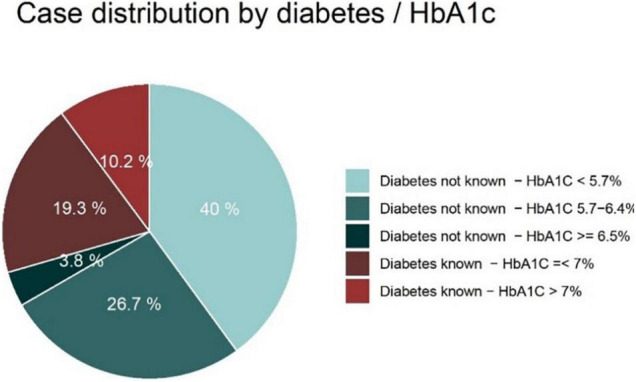
AMI cases (*n* = 2,317) by diabetes status and HbA1c values.

A total of 2,317 cases were included in this analysis. The distribution of cases by known diabetes and HbA1c group is displayed in Figure 1. Overall, 70.5% (*n* = 1,634) of patients had no type of previously known diabetes or disturbed glucose tolerance. Among these patients, 5.4% (*n* = 89) had HbA1c values of ≥ 6.5%. Another 37.9% (*n* = 619) of these patients had HbA1c values between 5.7 and 6.4%. A total of 683 patients (29.5%) had pre-known diabetes, of which 34.6% (*n* = 236) had HbA1c values of > 7%. Among patients in the diabetes group with HbA1c values of ≤ 7%, 4 patients (0.9%) had type 1 diabetes mellitus, 246 patients had type 2 diabetes mellitus (77.4%) and 87 patients had previously known disturbed glucose tolerance (19.5%); for another 10 patients (2.2%) there was no information on diabetes type. Among the patients in the diabetes group with HbA1c values of > 7%, 10 patients (4.2%) had type 1 diabetes mellitus, 220 patients had type 2 diabetes mellitus (93.2%), no patient had previously known disturbed glucose tolerance and for 6 patients (2.5%) there was no information on diabetes type available.

Table 1 displays the baseline characteristics according to diabetes and HbA1c. There were no significant sex-specific differences between the groups. The group with the lowest average age was the group of patients with no diabetes and an HbA1c < 5.7%; the highest average age was seen in the group with diabetes and HbA1c ≤ 7.0%. Diabetic patients were more often diagnosed with hypertension and hyperlipidemia, but were less likely to be current smokers. No significant differences could be observed regarding the occurrence of typical chest pain symptoms. Patients without diabetes were more likely to have a STEMI event. The frequency with which PCI was performed and the prescription of all four EBM at discharge was comparable among the groups. In the Supplementary Material we additionally provide information on statin and anti-diabetic medication use before the event.

**TABLE 1:** Baseline characteristics according to diabetes and HbA1c.

	No diagnosis of diabetes			Prevalent diabetes			
	HbA1c < 5.7% (*n* = 926)	HbA1c 5.7–6.4% (*n* = 619)	HbA1c ≥ 6.5% (*n* = 89)	HbA1C ≤ 7% (*n* = 447)	HbA1C > 7% (*n* = 236)	*P*-Value	*N*
Male	704 (76)	437 (70.6)	65 (73)	320 (71.6)	163 (69.1)	0.0785	2317
Age (mean, SD)	62.4 (12.8)	65.2 (12.5)	67.4 (11.2)	68.6 (10.8)	66.6 (11.3)	<0.001	2317
Glucose at admission (mg/dl)	119.0 (105–141)	131.0 (114–155)	165.0 (133–209.25)	161.5 (129.75–197)	235.0 (181–309)	<0.001	2300
HbA1c (%)	5.4 (5.2–5.5)	5.9 (5.8–6.1)	6.7 (6.5–6.9)	6.2 (5.8–6.6)	8.0 (7.4–9.2)	<0.001	2317
BMI (kg/m^2^)	26.0 (23.9–28.7)	27.1 (24.7–30.0)	28.7 (25.4–31.8)	28.0 (25.4–31.4)	29.4 (26.4–32.6)	<0.001	2147
Systolic blood pressure (mmHG)	140 (120–160)	140 (128–160)	144 (120–161.25)	143 (125–160)	140 (128.5–160)	0.5866	2291
Diastolic blood pressure (mmHG)	80 (68–92)	80 (70–90)	80 (67–92.5)	80 (67–90)	80 (68.25–88)	0.1666	2279
**Comorbidities**							
Hypertension	635 (68.6)	460 (74.3)	75 (84.3)	401 (89.7)	213 (90.3)	<0.001	2317
Hyperlipidemia	425 (45.9)	331 (53.5)	44 (49.4)	285 (63.8)	134 (56.8)	<0.001	2317
***Smoking status***						<0.001	2317
Current smoker	338 (36.5)	224 (36.2)	33 (37.1)	112 (25.1)	58 (24.6)	–	
Never smoker	250 (27)	160 (25.8)	22 (24.7)	146 (32.7)	65 (27.5)	–	
Ex-smoker	260 (28.1)	172 (27.8)	20 (22.5)	152 (34)	81 (34.3)	–	
No information	78 (8.4)	63 (10.2)	14 (15.7)	37 (8.3)	32 (13.6)	–	
**Clinical characteristics**							
Typical chest pain symptoms	803 (86.7)	530 (85.6)	73 (82)	384 (85.9)	186 (78.8)	0.0743	2317
Prehospital time in minutes	125 (79–314)	120 (77–255.75)	166 (69.75–459.75)	136 (74.5–330)	145 (89.5–421)	0.0514	2001
***Type of infarction***						0.0126	2317
STEMI	540 (58.3)	374 (60.4)	57 (64)	224 (50.1)	132 (55.9)	–	
NSTEMI	335 (36.2)	200 (32.3)	22 (24.7)	179 (40)	82 (34.7)	–	
Bundle branch block	39 (4.2)	33 (5.3)	7 (7.9)	33 (7.4)	18 (7.6)	–	
No information	12 (1.3)	12 (1.9)	3 (3.4)	11 (2.5)	4 (1.7)	–	
***Renal function according to GFR***						<0.001	2317
GFR ≥ 60 ml/min	703 (75.9)	397 (64.1)	51 (57.3)	256 (57.3)	128 (54.2)	–	
GFR 30–59 ml/min	196 (21.2)	197 (31.8)	34 (38.2)	158 (35.3)	89 (37.7)	–	
GFR < 30 ml/min	27 (2.9)	25 (4)	4 (4.5)	33 (7.4)	19 (8.1)	–	
Days in intensive care unit	2 (1–3)	2 (1–3)	2 (1–4)	2 (1–3)	2 (1–4)	0.4247	2298
**Laboratory value**							
Total cholesterol at admission (mg/dl)	202.5 (171–234)	203.5 (174–232.75)	189.0 (167.75–210.75)	184.0 (155.25–217.75)	198.0 (164–223.25)	<0.001	1082
Hemoglobin at admission (g/l)	143 (133–153)	142 (131–151)	143 (127–152)	139 (126.25–149)	142 (125–150.25)	<0.001	2315
Troponin I at admission (ng/ml)	0.605 (0.1–4.825)	0.595 (0.11–4.005)	0.600 (0.16–4.53)	0.570 (0.13–4.8)	0.875 (0.1425–7.7875)	0.7710	1955
Peak CRP levels (mg/l)	0.30 (0.29–0.77)	0.40 (0.29–0.955)	0.68 (0.33–2.46)	0.50 (0.29–1.375)	0.64 (0.29–2.05)	0.0861	2317
**Treatment**							
PCI	810 (87.5)	524 (84.7)	74 (83.1)	380 (85)	192 (81.4)	0.136	2317
Bypass therapy	95 (10.3)	78 (12.6)	14 (15.7)	50 (11.2)	40 (16.9)	0.0435	2317
Lysis therapy	9 (1)	6 (1)	0 (0)	1 (0.2)	2 (0.8)	0.6667	2317
Any revascularization therapy	873 (94.3)	587 (94.8)	85 (95.5)	420 (94)	220 (93.2)	0.8823	2317
**Medication at discharge**							
EBM*	725 (78.3)	473 (76.4)	64 (71.9)	339 (75.8)	173 (73.3)	0.3856	2317
Statins	827 (95.7)	532 (95.2)	73 (96.1)	382 (94.6)	196 (92.5)	0.3751	2115
Oral antidiabetics	2 (0.2)	2 (0.4)	3 (3.9)	167 (41.3)	132 (62.3)	<0.001	2115
GLP-1 receptor agonists	0 (0)	0 (0)	0 (0)	5 (1.2)	4 (1.9)	<0.001	2115
Insulin	2 (0.2)	2 (0.4)	4 (5.3)	60 (14.9)	106 (50)	<0.001	2115
**In-hospital complication**							
Any in-hospital complication	220 (23.8)	143 (23.1)	32 (36)	115 (25.7)	55 (23.3)	0.0993	2317
Cardiogenic shock	57 (6.2)	41 (6.6)	13 (14.6)	45 (10.1)	25 (10.6)	0.0028	2316
Pulmonary edema	29 (3.1)	20 (3.2)	3 (3.4)	23 (5.2)	15 (6.4)	0.0934	2315
Stroke	5 (0.5)	5 (0.8)	2 (2.2)	3 (0.7)	1 (0.4)	0.4309	2316
In-hospital reinfarction	8 (0.9)	8 (1.3)	4 (4.5)	9 (2)	4 (1.7)	0.0529	2317

*Antiplatelet drug, ACE blockers/ATII antagonist, beta-blockers, statins.

### Long-Term Mortality by Diabetes Status and HbA1c Values

**FIGURE 2:**
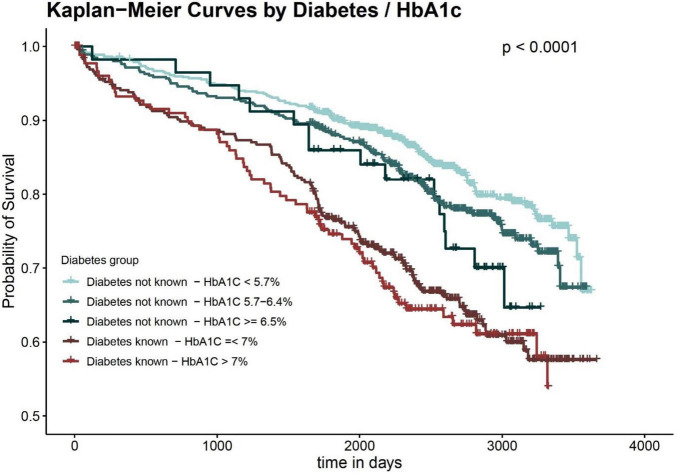
Long-term survival by diabetes status and HbA1c values.

Figure 2 shows the (unadjusted) Kaplan Meier curves according to known diabetes and HbA1c groups, and Table 2 displays the results of the COX regression models. Overall, the groups of patients without diabetes had distinctly better long-term survival than the two diabetes groups. Among patients without a diabetes diagnosis, patients with HbA1c values < 5.7% had a noticeable lower long-term mortality compared to the other two groups in the Kaplan Meier curve. This was confirmed by the COX regression models, which predicted a higher long-term mortality for the HbA1c 5.7–6.4%-group and the HbA1C ≥ 6.5%-group in comparison to the group with HbA1c values < 5.7%. However, the differences did not reach statistical significance in the adjusted models. The two diabetes groups, on the other hand, were significantly associated with higher long-term mortality in all COX models. In the fully adjusted model, they had comparable HR values (the group with HbA1C > 7% had marginally higher HR values).

**TABLE 2:** Results of the COX regression models.

ECG group	Unadjusted model		Adjusted for sex and age		Fully adjusted model*	
	HR [95% CI]	*p*-value	HR [95% CI]	*p*-value	HR [95% CI]	*p*-value
Diabetes not known HbA1c < 5.7%	1		1		1	
Diabetes not known HbA1c 5.7–6.4%	1.26 [0.97–1.64]	0.081	1.10 [0.85–1.43]	0.479	1.05 [0.79–1.38]	0.747
Diabetes not known HbA1c ≥ 6.5%	1.69 [1.02–2.81]	0.042	1.35 [0.81–2.24]	0.252	1.34 [0.77–2.31]	0.300
Diabetes known HbA1c ≤ 7.0%	2.23 [1.74–2.87]	<0.001	1.62 [1.26–2.09]	<0.001	1.59 [1.21–2.09]	0.001
Diabetes known HbA1c > 7.0%	2.48 [1.84–3.35]	<0.001	2.06 [1.53–2.79]	<0.001	1.73 [1.25–2.41]	0.001

*Adjusted for: sex, age, typical chest pain symptoms, diabetes, smoking, hyperlipidemia, hypertension, left-ventricular EF ≤ 30%, impaired renal function (according to eGFR), PCI, bypass surgery, lysis therapy, EBM, BMI, systolic blood pressure, and statin prescription at discharge.

### Prediction of Elevated HbA1c Levels Among Patients Without Known Diabetes

**FIGURE 3:**
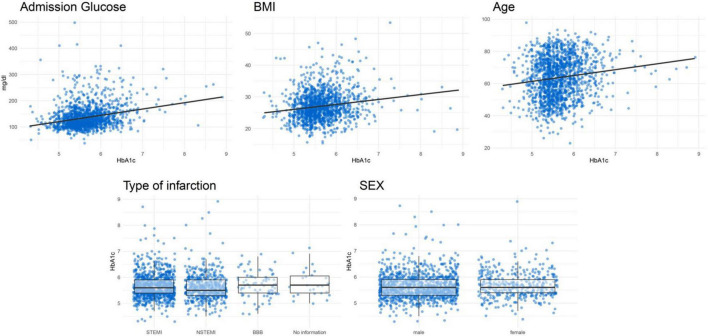
Correlation plots between HbA1c and continuous variables and boxplots for categorical variables for patients without known diabetes.

Figure 3 shows the association between certain important characteristics and HbA1c values in patients without known diabetes. Table 3 displays the results of several linear regression models with the dependent variable HbA1c. It shows a positive correlation for glucose at admission, BMI and age with HbA1c. No significant association was found for type of infarction and sex with HbA1c values. A final model, including all mentioned covariables, had an *R*^2^ of 11.08%.

**TABLE 3:** Linear regression models with the dependent variable HbA1c.

Variable	β-Estimation	*R* ^2^	*p*-value
Admission glucose	0.0028666	0.069	<0.001
BMI	0.01753	0.02734	<0.001
Age	0.0051029	0.01857	<0.001
Sex Male (reference/intercept) Female	5.63333 0.04844	0.00201	0.06999
Type of infarction STEMI (reference/intercept) NSTEMI Bundle branch block No information	5.65499 −0.03830 0.04374 0.11908	0.003307	0.1447
Combined model		0.1108 (Adjusted *R*^2^: 0.1067	<0.001

## Discussion

### High Frequency of Undiagnosed (Pre)diabetes in Acute Myocardial Infarction Patients

In the present study, we examined the frequency of elevated HbA1c values among AMI patients with and without known diabetes. HbA1c measurement is an appropriate tool to detect prediabetes and diabetes in general, and in AMI patients in particular, as it reflects average glucose levels over the previous 3 months (13–16). Of all patients without known diabetes, 43.3% had HbA1c values above 5.7%. This threshold is commonly used to distinguish between patients without risk of diabetes and patients with disturbed glucose tolerance or prediabetes (17). Prior studies reported diverging numbers for prevalence of (unknown) prediabetes in the general European population (18). The International Diabetes Federation (IDF), for example, suggested a prediabetes prevalence of about 5% in the European region (19), which is considerably lower than the prevalence we found for AMI patients. This is not surprising, as AMI patients have a higher risk profile with regards to many cardiovascular risk factors, including prediabetes and impaired glucose tolerance. Several prior studies reported a wide range of different results for prevalence of unknown (pre)diabetes among AMI patients (3–10). For the majority of studies, a comparison is difficult, as they were based on different study populations with different inclusion criteria (all AMIs, only STEMI events etc.), as well as different methods (HbA1c, OGTT, fasting glucose) and time points (at admission, at discharge) of determining prediabetes and impaired glucose tolerance. It can be assumed, that the results of the present study are very reliable, as they are based on data from a population-based registry with a high number of consecutive events included. Our results demonstrate that undetected disturbed glucose tolerance and prediabetes are widespread among a representative group of AMI patients.

### Undiagnosed (Pre)diabetes and Long-Term Outcome

In a subsequent analysis, we examined the associations between diabetes/HbA1c values and long-term mortality after AMI. Patients with known diabetes had significantly higher long-term mortality compared to the reference group (no known diabetes, normal HbA1c). This is not surprising, as diabetes is a well-known risk factor for AMI and a severe event (20). Nevertheless, among the diabetic patients, the high HbA1c group (> 7.0%) had similar long-term mortality compared to the low HbA1c group.

Among the patients without previously known diabetes, there were no significant differences regarding long-term mortality in the adjusted COX regression models. Nevertheless, a non-significant trend toward higher mortality was observed for patients with HbA1c > 5.7% compared to the reference group. In a prior study, Meier et al. conducted oral glucose tolerance tests in 129 AMI patients and compared long-term mortality in patients with impaired vs. normal glucose tolerance (7). They reported a significantly higher long-term mortality of AMI patients with impaired glucose tolerance compared to AMI patients without impaired glucose tolerance, diverging from the results of the present study, as we did not find a significant difference. In a further study (10), similar long-term mortality as that of the present study was obtained in a STEMI population, comparing subjects with unknown prediabetes (HbA1c 5.7–6.4%) to a normal HbA1c group. In contrast to the present study however, for subjects with unknown diabetes (HbA1c > 6.5%) an analogous 3-year mortality was observed compared to subjects with known diabetes.

One might expect a significantly higher mortality for patients with elevated HbA1c, but that is not what our data suggests. Different explanations for this should be considered. First, a moderately elevated HbA1c might not lead to increased long-term mortality among non-diabetic AMI patients, suggesting that physicians would not need to consider HbA1c levels as a risk factor for long-term mortality. An alternative explanation would be that there is indeed an association which did not reach significance in our study. Even though there was no significant difference in mortality for the first years after the event, it is possible that differences may not develop until a later time point, beyond our median follow-up period of 6.5 years. Most of the patients without known diabetes but with an elevated HbA1c might be in a very early phase of diabetes. As it can take several decades for serious microvascular complications of diabetes to manifest, it is impossible to detect these effects within a limited observational period. Annually, only 5–10% of all patients with prediabetes develop manifest diabetes mellitus, but up to 70% eventually do so within their lifetime (21). This affirms the assumption that the observational period of this study might have been insufficient to detect an increase in the long-term mortality of patients with prediabetes.

Another point to consider is that the outcome variable of this study was long-term mortality. Yet, there are many other adverse outcomes that can be of interest, e.g., myocardial reinfarction, heart failure, secondary mitral wave insufficiency or the progression of coronary artery disease. As those complications usually precede (cardiovascular) mortality, it can be assumed that we would have been able to detect differences in the HbA1c groups with regards to general adverse cardiovascular outcomes and major complications.

For these reasons, it still appears desirable that patients with elevated HbA1 values are identified early. In this way, education of patients (on diet, lifestyle changes), regular follow-up controls and, if necessary, pharmacological interventions (e.g., metformin) can be implemented in an early stage of the disease, which has been proven to reduce disease progression (21). The present study demonstrates that this issue does not affect only a few individual cases, but indeed concerns a considerable proportion of patients with AMI, and is therefore highly relevant.

### Characteristics and Factors Associated With Elevated HbA1c Values

Since HbA1c may not usually be determined in the context of an AMI, it raises the question of whether it would be useful and practical to routinely determine HbA1c values after AMI for the early detection of prediabetes. A more favorable solution may be to identify factors that predict an increased risk of elevated HbA1c values. In this way, physicians might only need to measure HbA1c values in patients with a high risk of elevated HbA1c levels. We attempted to identify risk factors and characteristics that would potentially predict an increased risk of elevated HbA1c values by calculating linear regression models for the predictor variables glucose at admission, BMI, age, sex and type of infarction. Even though BMI, age and admission glucose were significantly associated with HbA1c levels, their predictive abilities (*R*^2^ values) were very limited. Even in a final model including all five predictor variables, the *R*^2^ was only 11.08%, which is certainly not sufficient for an adequate risk stratification at the acute event.

### Strengths and Limitation

The present study is characterized by some particular strengths. First to mention is the large sample size, the consecutive enrollment of patients and long follow-up period with a median time of 6.5 (IQR: 4.9–8.1) years. Standardized data collection was performed by conducting patient interviews during the hospital stay and via patient chart review. Nevertheless, there are also some limitations. Since only patients between 25 and 84 years were included, results cannot be applied to older age-groups, and findings may not be generalized to all ethnic groups. As this is an observational study, causality cannot be proven. Furthermore, it is possible that some confounders, which could have influenced the associations, were not available and could therefore not be included in the analysis. Thus, residual confounding cannot be entirely excluded. Likewise, we might not have considered some valuable predictors of increased HbA1c values in patients without known diabetes.

## Conclusion

About 30% of AMI patients have a previously known diagnosis of diabetes, and these patients have a significantly higher risk of earlier death as compared to non-diabetics. In addition, 5.4% of AMI patients without known diabetes have HbA1c values ≥ 6.5%. A further 37.9% have HbA1c values between 5.7 and 6.4%, which demonstrates that undiagnosed (pre)diabetes is a widespread issue among AMI patients. However, in this study we could not identify significant differences between HbA1c groups and long-term mortality in non-diabetic AMI patients. Nonetheless, it remains important to detect derangements of glucose metabolism in these patients at an early stage. Over the course of several years it is quite likely for these patients to develop a manifest diabetes mellitus disease and as a consequence a number of serious long-term complications. By identifying such patients, physicians have a good chance of early intervention (education, lifestyle changes, or medication) and consequent prevention of diabetes-associated complications or even of the manifestation of diabetes mellitus in the first place.

## Data Availability Statement

The datasets generated during and/or analyzed during the current study are not publicly available due to data protection aspects but are available in an anonymized form from the corresponding author on reasonable request.

## Ethics Statement

This study involves human participants and was reviewed and approved by Ethics Committee of the Bavarian Medical Association (Bayerische Landesärztekammer). The patients/participants provided their written informed consent to participate in this study.

## Author Contributions

TS and CM conceived the study. TS performed the statistical analysis and drafted the manuscript. CM supervised data analysis and was responsible for the acquisition of the data. EH, MH, AP, and JL contributed to data acquisition and revised the manuscript. All authors approved the final manuscript.

## Conflict of Interest

The authors declare that the research was conducted in the absence of any commercial or financial relationships that could be construed as a potential conflict of interest.

## Publisher’s Note

All claims expressed in this article are solely those of the authors and do not necessarily represent those of their affiliated organizations, or those of the publisher, the editors and the reviewers. Any product that may be evaluated in this article, or claim that may be made by its manufacturer, is not guaranteed or endorsed by the publisher.
