# Supplementary material for: Undiagnosed Impaired Glucose Tolerance and Type-2 Diabetes in Acute Myocardial Infarction Patients: Fequency, Characteristics and Long-Term Mortality
Source: Front Cardiovasc Med. 2022 Apr 25;9:869395. doi: 10.3389/fcvm.2022.869395 (PMC9081974; doi:10.3389/fcvm.2022.869395)
Supplement: Supplementary file 1 [file Table_1.docx]

***Supplementary material***

**Title:** Undiagnosed impaired glucose tolerance and type-2 diabetes in acute myocardial infarction patients: frequency, characteristics and long-term mortality

**Table 1:** M*edication before the event according to diabetes and HbA1c*

|  | ***No diagnosis of diabetes*** | | | ***Prevalent diabetes*** | |  |  |
| --- | --- | --- | --- | --- | --- | --- | --- |
|  | ***HbA1c <5.7%***  ***(n=926)*** | ***HbA1c***  ***5.7-6.4%***  ***(n=619)*** | ***HbA1C***  ***≥ 6.5%***  ***(n=89)*** | ***HbA1C***  ***≤ 7%***  ***(n=447)*** | ***HbA1C***  ***> 7%***  ***(n=236)*** | ***P-Value*** | ***N*** |
| statins | 78 (16.5) | 58 (19.7) | 11 (22.9) | 87 (35.2) | 34 (26.6) | <0.001 | 1192 |
| oral antidiabetics | 1 (0.2) | 1 (0.3) | 2 (4.2) | 101 (40.9) | 78 (60.5) | <0.001 | 1193 |
| GLP-1 receptor agonists | 0 (0) | 0 (0) | 0 (0) | 3 (1.2) | 1 (0.8) | <0.001 | 1192 |
| insulin | 0 (0) | 1 (0.3) | 2 (4.1) | 32 (12.9) | 48 (37.2) | <0.001 | 1195 |
